# Physical Conditions Prevailing in the Nasal and Maxillary Sinus Cavities Based on Numerical Simulation

**DOI:** 10.3390/medicina59061094

**Published:** 2023-06-05

**Authors:** Monika Morawska-Kochman, Ziemowit Miłosz Malecha, Krzysztof Zub, Jakub Kielar, Krzysztof Dudek, Kamil Nelke, Tomasz Zatonski

**Affiliations:** 1 Department of Otolaryngology, Head and Neck Surgery, Medical University, Borowska 213, 50-556 Wroclaw, Poland; monika.morawka-kochman@umw.edu.pl (M.M.-K.); tomasz.zatonski@umw.edu.pl (T.Z.); 2 Department of Cryogenics and Aerospace Engineering, Wroclaw University of Science and Technology, Wybrzeze Wyspianskiego 27, 50-370 Wroclaw, Poland; jakub.kielar@pwr.edu.pl; 3 Statistical Analysis Centre, Wroclaw Medical University, 50-368 Wroclaw, Poland; krzysztof.dudek@pwr.edu.pl; 4 Maxillofacial Surgery Ward, EMC Hospital, Pilczycka 144, 54-144 Wroclaw, Poland; 5 Health Department, Academy of Applied Sciences, Academy of Silesius, Zamkowa 4, 58-300 Walbrzych, Poland

**Keywords:** airflow, nasal cavity, sinus cavity, computational fluid dynamics, temperature, humidity, microorganisms

## Abstract

*Background and Objectives*: This paper presents a unique study that links the physical conditions in the nasal passage with conditions that favour the development of bacterial strains and the colonization of the mucous membranes of the nose and paranasal sinuses. The physical parameters considered were air flow, pressure, humidity, and temperature. *Materials and Methods*: Numerical models of the human nose and maxillary sinus were retrospectively reconstructed from CT images of generally healthy young subjects. The state-of-the-art numerical methods and tools were then used to determine the temperature, humidity, airflow velocity, and pressure at specific anatomical locations. *Results*: The results were compared with optimal conditions for bacterial growth in the nose and sinuses. *Conclusions*: Temperature, humidity, air velocity, and pressure were shown to play critical roles in the selection and distribution of microorganisms. Furthermore, certain combinations of physical parameters can favour mucosal colonisation by various strains of bacteria.

## 1. Introduction

The question of the development and role of paranasal sinuses in humans remains open. The most popular theories come from three hypotheses: structural, evolutionary, and functional. The structural hypothesis assumes that sinus cavities reduce the weight of the face and relieve the muscles of the head and neck, optimizing balance. Evolutionary theory claims that its development resulted from adapting to maintaining airways outside the aquatic environment. Finally, the functional hypothesis assumes that paranasal sinuses are a voice resonator, thermal insulator, and protector against brain injury and increase the surface area of the mucous membrane that humidifies inhaled air [1,2,3,4].

The anatomical variability of the shape and volume of the nasal cavity and sinuses affects the prevailing physical conditions. Factors such as temperature, humidity, airflow, and air pressure can simultaneously play a significant role in their proper functioning [5]. They can also indirectly shape the selection and distribution of microorganisms on the mucous membrane [6]. The available literature lacks research results describing the relationships between physical conditions and environmental requirements of bacteria colonizing the nasal cavity and sinuses. The use of computer modelling techniques have proved to be helpful. Three-dimensional geometric modelling has opened up new possibilities for physical and digital airflow simulations in the nasal cavity based on anatomically precise computer models [7]. Measurements based on computational fluid dynamics (CFD) have been the subject of studies demonstrating the method’s effectiveness [8,9,10,11]. Numerical analysis has been used in various studies such as airflow diagnostics in patients with sleep apnoea, deposition fraction, and drug delivery efficiency [12,13]. Recent research has confirmed that the use of numerical modelling to analyse airflow through the nasal cavity (virtual rhinomanometry) can be considered a reliable method [14]. Many studies have shown that simulations and numerical modelling, particularly numerical fluid dynamics (CFD), are mature and reliable enough to be treated as part of personalized medicine. Computational fluid dynamics (CFD) has been a valuable diagnostic tool used in numerical simulations and to analyse data related to the interactions of liquid and gas particles whose movements are also limited by solid surfaces, to evaluate heat and moisture exchange [15,16]. Several studies have investigated the use of CFD in paranasal sinus modelling, e.g., to investigate the effect of nasal anatomical deformations on airflow [17] or to simulate the distribution of inhaled particles within the sinuses [18]. The CFD method has also been used to study air flow, and heat and moisture exchange in healthy and diseased noses and sinuses after surgical procedures or to investigate intranasal medicine deposition [16,19,20,21,22,23]. In addition, as stated in the work [24], numerical simulations may become part of personalized medicine, especially when appropriate software is developed to shorten the tedious calculations.

Evolution has led to complex relationships between microorganisms that form ecosystems and their specific host organism [25]. Due to the different environmental requirements, their distribution varies between regions of the human body. For example, the upper respiratory tract provides suitable conditions for the colonisation of bacteria, which also depend on various external factors (such as temperature and humidity) and internal factors (such as diseases or immune responses) [26].

Summarizing the above review of the literature, it can be stated that numerical calculations can be a reliable and detailed source of information on the physical conditions prevailing in the nasal passage. It allows researchers to analyse a wide spectrum of data from anywhere in the nasal cavity and paranasal sinuses without performing invasive and uncomfortable in vivo procedures. On the other hand, a thorough knowledge of these conditions can help to better understand the formation of conditions favourable for the development of bacterial colonies and infections.

The aim of this study was to determine and compare, using state-of-the-art CFD calculation methods, the values of temperature, humidity, airflow velocity, and air pressure in healthy individuals in three clinically significant locations: the inferior nasal meatus, the area near the opening of the ostiomeatal complex, and the maxillary sinus cavity. To our knowledge, no study published so far has simultaneously considered all clinically significant characteristics of nasal airflow, such as pressure, humidity, velocity and temperature, at the locations investigated in the present study. Moreover, no study to date has attempted to link these flow characteristics, obtained on the basis of calculations, with the requirements of bacterial strains colonizing the mucous membranes of the nose and paranasal sinuses.

## 2. Material and Methods

The presented study utilized Computer Tomography (CT) images to reconstruct realistic human nose and maxillary sinus models. The CT images comprised 100 cross-sections, each with a 512 × 512 pixels resolution in DICOM format. Ten young and generally healthy subjects (four women and six men) were retrospectively analysed (mean age = 27 years; SD = 3.46; min. = 23; max. = 34). All individuals included in the analysis never reported nasal or sinus complaints and showed no imaging evidence of sinusitis. The CT examinations were performed previously as part of the preparation for orthognathic surgery. Therefore, the inclusion criteria were patients without symptoms and signs of sinonasal mucosal inflammation selected for orthognathic surgery treatment. Exclusion criteria consisted of patients under 18 years of age and older than 35 years of age, with acute or chronic rhinosinusitis, with previous nose/sinus surgery, with allergic rhinitis or smokers. The patients should not be on medication for one month before the CT scan. The study was approved by the Bioethics Committee at the local Medical University and conducted according to the principles of the Declaration of Helsinki (KB-545/2015).

After the reconstruction of the ethmoid labyrinth, the sphenoid and frontal sinuses were bilaterally excluded from the computation, and calculations were performed for the two most extensive maxillary sinuses. Based on anatomical and clinical considerations, three locations were selected for analysis: the inferior nasal duct, the area around the issue of the ostiomeatal complex, and the maxillary sinus floor. These sites were chosen due to the routine collection of swab cultures for microbiological testing [27,28,29].

## 3. Numerical Simulation Methods

**Figure 1:**
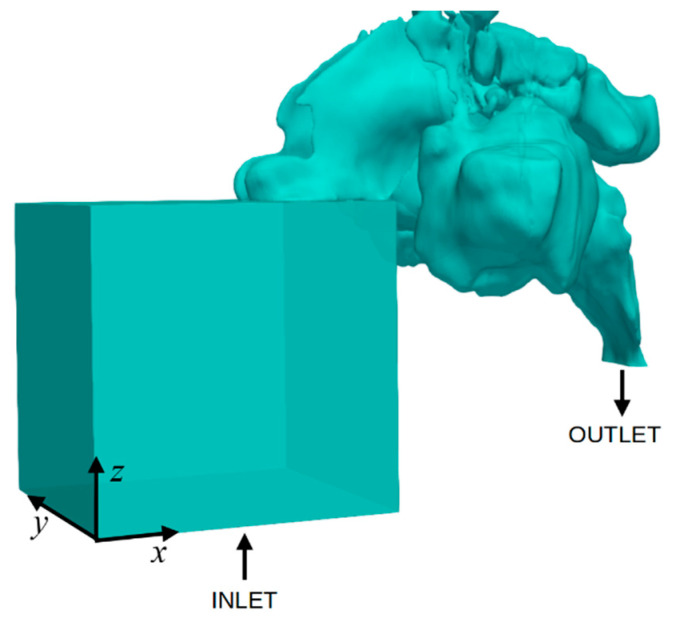
The computational domain of one of the cases consists of the nasal cavity reconstructed from CT images and a box simulating the outside environment.

To maintain natural airflow entering the nasal cavity and ensure the numerical stability of the model, an additional computational area was created that was connected to the nasal openings. This area was shaped like a 60 mm × 60 mm × 65 mm cuboid and simulated the external environment, as shown in Figure 1. This approach has also been used in other papers [30]. In addition, an additional 50 mm extension piece was attached to the end of each model’s nasopharyngeal section to ensure numerical stability and prevent flow reversal at the nasopharyngeal outlet.

Following the reasoning presented in a series of papers regarding the modelling of airflow in the nasal cavity [30,31,32,33], it was assumed that the flow was incompressible, laminar, and steady:

(1) 𝛻⋅u=0

(2) $$𝛻\cdot \left(\rho uu\right)=-𝛻p+𝛻\cdot \mu 𝛻u$$

(3) $$\rho C_{p}\left(u\cdot 𝛻T\right)=k{𝛻}^{2}T$$

(4) $$u\cdot 𝛻C=D{𝛻}^{2}C$$

The velocity vector is represented by $u=\left(u_{x},u_{y},u_{z}\right)$, while *p* represents the pressure in Pa, *T* represents temperature, *C* represents the concentration of water vapour, *ρ* represents the density, *μ* represents the dynamic viscosity, *C_p_* represents the specific heat, and *k* represents the thermal conductivity of air. Finally, *D* represents the mass diffusion coefficient of the water vapour into the air.

Equation (1) is the principle of mass conservation; Equation (2) is the Navier–Stokes equation for incompressible fluids and describes the principle of conservation of momentum; Equation (3) is the principle of conservation of energy in the flow; and Equation (4) describes the process of diffusion and transportation of moisture in the flow.

Similarly, as in [9,30], a two-film theory was adopted to determine the mass fraction of water in the nasal cavity. Therefore, the transport of vapour from the walls of the nasal cavity to the interior was simulated using the following boundary condition.

(5) $$-D\;\frac{\partial C}{\partial n}=\frac{C_{a}-C_{b}}{R}$$

In Equation (5), *C_a_* = 0.032 represents the mass fraction of the vapour at the interface between the organ and mucous (ambient mass fraction of the vapour), and n indicates a normal direction towards the nearest wall. The value of *C_a_* is constant because the mucous is constantly supplied with water by the capillary layer, *C_b_* is the mass fraction of vapour on the surface of the nasal cavity wall, and *R* is the cumulative mass diffusion resistance:

(6) $$R=\frac{D_{bl}}{\delta_{bl}}+\frac{D_{memb}}{\delta_{memb}}$$

$D_{bl}=3\cdot 10^{-5}$ m^2^/s and $D_{memb}=2.6\cdot 10^{-5}$ m^2^/s are the mass diffusion coefficient of the boundary layer and mucous membrane [14]. The thickness of the boundary layer and the mucous membrane layer thickness is $\delta_{bl}=0.3$ mm and $\delta_{memb}=0.5$ mm, respectively [34].

Equations (1) through (4) were discretised using the finite volume method (FVM) and solved numerically using the OpenFOAM (Open Source Field Operation and Manipulation) software [35] along with the SIMPLE (Semi-Implicit Method for Pressure-Linked Equations) algorithm [36,37]. Table 1 shows the boundary conditions used in the created numerical model illustrated in Figure 1. All boundaries of the box simulating the external environment, except for the inlet, are called “box walls”, and the outer boundaries of the nasal cavity are called “nasal walls”.

Table 2 displays the information regarding the numerical grid used in the simulations. The grid was composed primarily of hexagonal and polyhedral cells, with a total of approximately 6 million grid cells, consistent with the quantity utilized in previous numerical grid studies [30]. In addition, the mesh cells were progressively reduced in size near the walls to ensure an accurate representation of the intricate shape of the nasal cavity wall.

**Figure 2:**
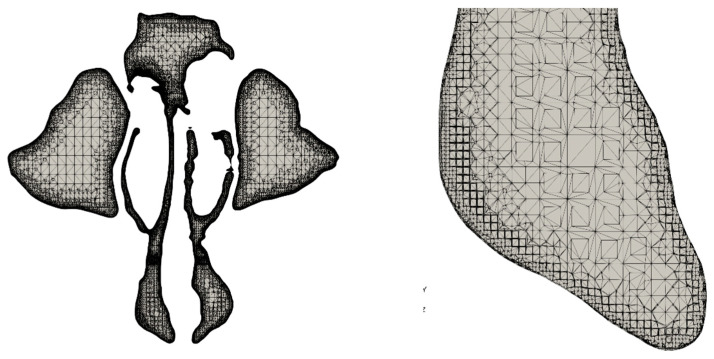
An example of the numerical mesh structure. (**Left**) Cross-section in the *y-z* plane just above the nostrils; (**Right**) enlarged section of inlet into the right nostril.

Figure 2 presents a cross-section through the calculation domain from Figure 1 and shows the structure of the numerical grid. It can be seen that the mesh very closely reproduces the actual shape of the nasal cavity, and the mesh cells became smaller as they neared the wall. In addition, the narrow channels contained a sufficiently large number of grid cells to represent the flow profile. These features ensure the physical behaviour of the flow and heat and mass transfer at the walls and reliable calculation results.

## 4. Results

**Figure 3:**
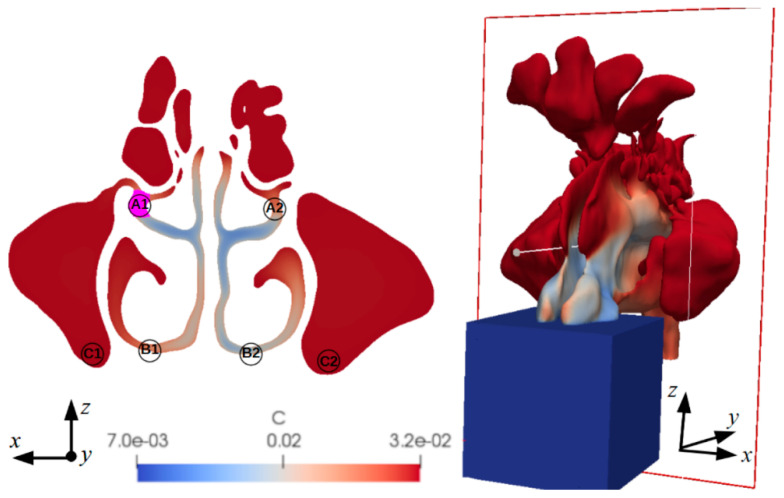
(**Left**) Cross-section through the nostrils indicating the measurement locations: A1, A2—the area around the ostiomeatal complex, B1, B2—the inferior nasal duct, C1, C2—maxillary sinus floor. (**Right**) General view of the 3D geometry of the nostrils with a sectional plane. The colour map indicates the humidity (C) in the nostrils.

Figure 3 shows example computational results of the humidity in the nasal cavity (of one of the considered geometries reproduced from the CT images), along with the selected measurement locations for further analysis: the area around the ostiomeatal complex (A1, A2), the inferior nasal duct (B1, B2), and the maxillary sinus floor (C1, C2). It was ensured that the measurement locations on the left and right sides of the nasal cavity were as anatomically similar as possible. It can be seen that the mass fraction of the vapour in the area representing the external environment was 0.007%. After air flows into the nasal passage, its humidity increased and in places where the flow is very low or close to zero (maxillary sinuses), the mass fraction of the vapour in the air increased to 0.032%, which corresponds to the vapour content in the mucous membranes.

Table 3 presents the temperature and humidity, and Table 4 presents the mean velocity and pressure magnitude, in the selected locations. It can be observed that the remaining differences between the nose’s right and left sides were insignificant. A comparison of individual parameters in different areas showed that the *p*-value was greater than 0.05. Consequently, the averaged values were used in further analyses and discussions.

**Figure 4:**
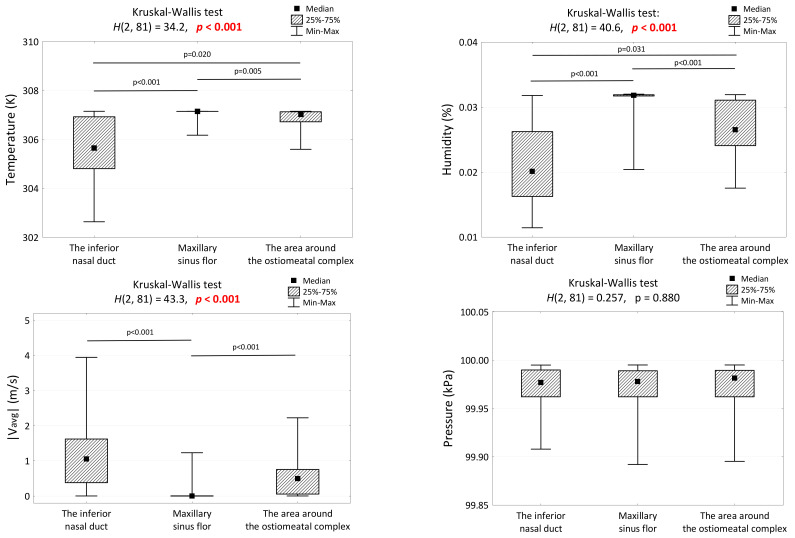
Comparison of temperature, humidity, flow velocity, and pressure values at selected locations for the nose and sinuses.

Figure 4 presents the comparison of temperature, humidity, flow velocity, and pressure values at the selected locations in the nose and sinuses. It should be noted that the obtained empirical distributions of the variables significantly deviated from the theoretical normal distribution (verified using the Shapiro–Wilk test). Therefore, the results shown in Figure 4 are presented as the median (Me) and the interquartile range IQR (Q1 and Q3). Nonparametric tests (Wilcoxon, Kruskal–Wallis, and Friedman) were used to compare the average values between the groups.

Analysing the results from Figure 4, it can be seen that statistically significant differences were observed in the three examined locations for all evaluated parameters except for pressure, which had similar values at every location. The highest air temperature and humidity were recorded in the maxillary sinus lumen and the lowest in the inferior nasal meatus. The air flow had almost zero velocity at the bottom of the maxillary sinus.

## 5. Discussion

The nasal cavity connects the external environment, full of various pollutants and of variable physical parameters, with the upper and lower respiratory tract’s protected and regulated internal environment. The primary functions of the nasal cavity are to transport, clean, heat, and humidify inhaled air. Disorders of airflow through the nose caused by anatomical anomalies can alter the regulation of heat and moisture, which is believed to harm the physiology of the respiratory tract, predisposing to, among others, sinus inflammatory conditions [3]. The question arises as to whether modified physical conditions at different locations will favour the colonisation of pathogenic bacteria with requirements that differ from those of physiological flora.

The most clinically significant locations should be selected to determine the conditions conducive to bacterial colonization in the nose and sinuses. The preferred sampling sites include the anterior nares, middle meatus, nasopharynx, and occasionally the sinuses themselves [38,39,40]. The middle meatus is the most commonly chosen site. With the introduction of FESS, swabs have replaced sinus puncture aspirates as the standard sampling technique [41]. However, for the sinus microflora, a mid-meatus swab may not be as representative as is currently believed [42]. The results of this research do not accurately reflect the local sinus microbiome, and the data from the literature do not provide a uniform opinion about the microbiota. According to Yan et al., there are microenvironment-dependent interactions between *Corynebacterium accolens*, *C. pseudodiphtheriticum*, and *S. aureus*. They suggest that humidity is a favourable factor for the *Corynebacterium* and *Staphylococcus* species and may explain their abundant presence in the nasal mucosa [43].

Comparing swabs from the inferior and middle nasal meatuses in the same patient showed significant individual variability in the microbiota and similar intrapersonal profiles. Furthermore, the middle nasal meatus had a lower microbiological diversity in the same patient than the inferior nasal meatus [38]. Recently, a so-called “core microbiome” has been identified for the mucous membrane of the maxillary sinuses. Although the individual microbiome is variable, it usually consists of species such as *Corynebacterium*, *Staphylococcus*, *Streptococcus*, *Haemophilus*, and *Moraxella*. Comparing the results of microbiological studies from the nasal mucosa and the interior of the maxillary sinuses, a statistically significant correlation was found between the type and location of eight microorganisms, especially *Propionibacterium acnes*, which was identified only in the sinus cavities [42,44].

We measured the temperature, humidity, air velocity, and air pressure in three locations in reference to our earlier studies [45]. The average air temperature between 32 and 34 °C did not differ statistically between the right and left sides. The lowest temperature was found on the floor of the nasal cavity, while the highest was in the lumen of the sinus. However, it was similar to that of the ostiomeatal complex region. Although the differences were statistically significant, the temperature never exceeded 34.05 °C.

Similarly, Zang et al.’s calculations indicate higher maxillary sinus temperatures values than those of the middle nasal meatus. This phenomenon is explained by the low airflow velocity through the sinus, which prolongs its contact time with the mucosa [46]. The calculation results are consistent with the in vivo measurements conducted by Lindeman et al. In the inferior meatus, at the anterior edge of the inferior turbinate, they obtained values ranging from 30.2 to 32.2 °C [47]. Similar results were presented by Keck et al. for healthy individuals. The temperature was measured with a miniaturised thermocouple in the nasal vestibule, at the nasal valve, in the anterior part of the middle turbinate, and in the nasopharynx. A logarithmic increase in air temperature was observed from the anterior nostril to the nasopharynx, where it reached about 34 °C [48]. Therefore, our numerical calculations based on CT scans correspond to the values obtained from in vivo studies.

The temperature values impact the bacterial flora on the mucous membrane, as shown in Table 5. A good example is bacteria of the *Streptococcus* genus, which colonize the respiratory tract and are subject to the effects of varying temperature, oxygen, and pressure levels depending on their location. At an average temperature of around 33 °C in the nasopharynx, they remain part of the physiological flora. During viral infections and when the tissue barrier is breached, the temperature to which they are exposed increases to 37 °C or greater [49]. In the work of Tóthpál et al., the effect of temperature and oxygen level changes on the growth of different strains and serotypes of *Streptococcus pneumoniae* was studied. They grew at the highest density and had the shortest lag phase under conditions similar to the nasopharynx temperature, that is, around 30 °C, rather than under laboratory conditions of around 37 °C.

In our calculations, the temperature for all three locations did not exceed 34 °C, which was therefore optimal for most bacteria colonising the nasal mucous membrane and paranasal sinuses. Of course, in addition to an increase in temperature during an infection, many other factors, such as different oxygen levels or increased mucus production, also play an important role. Following endoscopic procedures, *Staphylococcus aureus* is the most commonly identified bacterium [50], whose optimal growth temperature ranges between 30 and 37 °C. Therefore, the question arises as to whether a moderate decrease in temperature, due to the broader opening of the sinuses, would enhance the colonization of this bacterium on the mucous membrane.

Another physical factor that we investigated was air humidity. It was the lowest in the inferior nasal meatus and the highest in the maxillary sinus floor, probably due to almost zero airflow through its lumen. However, the differences were not statistically significant. In the results presented by Keck et al., the measured humidity and error ranges were plotted. Both in vivo and simulated values showed the same trend: the relative humidity increased rapidly by 2.5 cm from the anterior nostrils. Then, it rose slowly, but the inhaled air was humidified to nearly 100% [48].

The airflow velocity in the maxillary sinuses is significantly lower than in the nasal meatuses [46,51]. Our research confirmed this finding, although the differences were not statistically significant. Developmental anomalies, such as a deviated nasal septum, can change the airflow pattern, but it has not been proven that this can affect, for example, heating [52].

In our study, based on examination results from healthy people, the pressure differences between the interior of the sinus, the inferior nasal meatus, and the ostiomeatal complex were not statistically significant. According to Xiong et al. [51], the air pressure is simultaneously high in the anterior part of the inferior and middle nasal meatuses and the uncinate process (ostiomeatal complex). As in our study, a slight pressure difference was observed between the interior of the maxillary sinus and the nasal meatuses, allowing for perceptible air exchange [51]. At the same time, it should be emphasized that various biological factors always come into play, including symbiosis or inter-species competition [53,54].

The limitation of our work was the small group of healthy subjects who were referred for CT scans. All patients were qualified only for cosmetic orthognathic surgery. In addition, the calculations performed for one person were quite time consuming and it would be necessary to prepare calculation applications to facilitate the work.

In summary, CFD modelling methods can provide reliable information about the anatomical structure and physical conditions in the nose and paranasal sinuses. However, it can also be the basis for studying their relationships and the bacterial flora inhabiting this section of the respiratory tract. The study confirmed that different conditions prevail in the nasal and sinus cavities (in other locations). It can be assumed that a chronic inflammatory process that causes oedema and hypertrophy of the mucous membrane will change the parameters of airflow. This will also affect the humidity and temperature, which may be favourable or unfavourable for the growth of certain bacteria. In fact, this cannot be the only significant factor in the complex chronic inflammatory process of the sinonasal mucous membrane. However, this method may contribute to the precise treatment planning for chronic rhinosinusitis.

## 6. Conclusions

The main findings of the presented study can be summarized as follows:

- Physical conditions may promote the colonisation of the mucous membrane by various bacterial strains.
- The CFD method enables the determination of airflow, heat exchange, and humidity values in the nose and sinuses, and the results are consistent with other in vivo studies.
- These findings highlight the variations in air temperature, humidity, and airflow patterns within the nasal cavity, specifically emphasizing the distinct characteristics of the maxillary sinus and the inferior nasal meatus.
- Our study may initiate further exploration of the relationship between nasal and sinus conditions and the requirements of colonising bacteria.

## Figures and Tables

**Table 1:** Boundary conditions used in the numerical calculations of the model illustrated in Figure 1. “Nasal wall” corresponds to all the walls of the nasal cavity, “Box walls” correspond to the boundaries of the external environment (apart from the “Inlet” boundary).

Physical Conditions	Inlet	Outlet	Nasal Wall	Box Walls
Velocity, l/min	15	15	No-slip	slip
Temperature, °C	25	36.6	34	25
Vapour, %	0.007	$\frac{\partial \;C}{\partial n}=0$	Equation (5)	0.007
Pressure, Pa	100,000	$\frac{\partial \;p}{\partial n}=0$	$\frac{\partial \;p}{\partial n}=0$	$\frac{\partial \;p}{\partial n}=0$

**Table 2:** Resolution and shape details of the computational mesh used in the study.

Number of Mesh Cells and Their Shape			Min Volume of Cell	Max Volume of Cell
hexahedra	polyhedra	total number	m^3^	m^3^
~4 million	~2 million	~6 million	$∼10^{-13}$	$∼10^{-8}$

**Table 3:** Humidity and temperature of the air flowing through three selected anatomical locations.

Location	Side	Temperature (°C)	Humidity (%)
Inferior nasal meatus	Right	33.15 (32.35 ÷ 33.95)	0.022 (0.018 ÷ 0.031)
	Left	32.05 (31.05 ÷ 33.75)	0.019 (0.015 ÷ 0.024)
	R vs. L	*p*-value = 0.126	*p*-value = 0.094
Ostiomeatal complex	Right	33.85 (33.55 ÷ 33.95)	0.027 (0.026 ÷ 0.031)
	Left	33.65 (33.65 ÷ 33.95)	0.026 (0.022 ÷ 0.028)
	R vs. L	*p*-value = 0.827	*p*-value = 0.275
Maxillary sinus floor	Right	34.05 (34.05 ÷ 34.05)	0.032 (0.032 ÷ 0.032)
	Left	34.05 (34.05 ÷ 34.05)	0.032 (0.032 ÷ 0.032)
	R vs. L	*p*-value = 0.827	*p*-value = 0.716

**Table 4:** The magnitude of velocity and the pressure of air in three selected anatomical locations.

Location	Side	|V_avg_|(m/s)	Pressure (Pa)
Inferior nasal meatus	Right	0.512 (0.127 ÷ 1.140)	99,978 (99,946 ÷ 99,990)
	Left	1.258 (1.056 ÷ 2.165)	99,975 (99,973 ÷ 99,990)
	R vs. L	*p*-value = 0.062	*p*-value = 0.846
Ostiomeatal complex	Right	0.491 (0.031 ÷ 0.646)	99,981 (99,962 ÷ 99,989)
	Left	0.542 (0.092 ÷ 0.857)	99,982 (99,966 ÷ 99,989)
	R vs. L	*p*-value = 0.320	*p*-value = 0.903
Maxillary sinus floor	Right	0.000 (0.000 ÷ 0.002)	99,980 (99,961 ÷ 99,989)
	Left	0.000 (0.000 ÷ 0.000)	99,974 (99,963 ÷ 99,988)
	R vs. L	*p*-value = 0.942	*p*-value = 0.961

**Table 5:** Optimal temperature values for the growth of selected bacterial strains identified on the mucous membrane of the nose and sinuses, based on the published literature.

Bacterial Strain	Oxygen Requirements	Optimal Growth Temperature Ranges	Reference(s)
*Streptococcus* spp.	aerobic	35–37 °C, maximal grow at low temperatures (~33 °C)	[45,50]
*Corynebacterium* spp.	anaerobic, aerobic, and microaerophilic	31–37 °C	[46]
*Propionibacterium* spp.	anaerobic to aerotolerant	25–35 °C thermotolerant	[47]
*Staphylococcus* spp.	aerobic or facultatively anaerobic	30° to 37 °C	[48]
*Haemophilus* spp.	aerobic or facultatively anaerobic	35–37 °C	[49]
*Moraxella* spp.	aerobic	33–35 °C	[51]

## Data Availability

Data sharing is not applicable to this article.
